# *APOE* genotype and sex modulate Alzheimer’s disease pathology in aged EFAD transgenic mice

**DOI:** 10.3389/fnagi.2023.1279343

**Published:** 2023-10-31

**Authors:** Deebika Balu, Ana C. Valencia-Olvera, Zarak Islam, Clare Mielczarek, Allison Hansen, Tamara M. Perez Ramos, Jason York, Mary Jo LaDu, Leon M. Tai

**Affiliations:** ^1^Department of Anatomy and Cell Biology, University of Illinois at Chicago, Chicago, IL, United States; ^2^University of Illinois College of Medicine, Chicago, IL, United States; ^3^University of Illinois College of Medicine, Peoria, IL, United States; ^4^School of Medicine, St. George’s University, St. George’s, Grenada

**Keywords:** Alzheimer’s disease, *APOE4*, female risk, transgenic mice, amyloid-beta

## Abstract

Increasing evidence supports that age, *APOE* and sex interact to modulate Alzheimer’s disease (AD) risk, however the underlying pathways are unclear. One way that AD risk factors may modulate cognition is by impacting amyloid beta (Aβ) accumulation as plaques, and/or neuroinflammation Therefore, the goal of the present study was to evaluate the extent to which age, *APOE* and sex modulate Aβ pathology, neuroinflammation and behavior *in vivo*. To achieve this goal, we utilized the EFAD mice, which express human *APOE3* or *APOE4* and have five familial AD mutations (FAD) that result in Aβ42 overproduction. We assessed Aβ levels, reactive glia and Morris water maze performance in 6-, 10-, 14-, and 18-month-old EFAD mice. Female *APOE4* mice had the highest Aβ deposition, fibrillar amyloid deposits and neuroinflammation as well as earlier behavior deficits. Interestingly, we found that female *APOE3* mice and male *APOE4* mice had similar levels of pathology. Collectively our data support that the combination of *APOE4* and female sex is the most detrimental combination for AD, and that at older ages, female sex may be equivalent to *APOE4* genotype.

## 1. Introduction

Alzheimer’s disease (AD) is a progressive neurodegenerative disease associated with deficits in the cognitive system (Warren et al., 2023). Important risk factors for sporadic AD include age, and sex (Riedel et al., 2016). Age is the greatest overall AD risk factor, with 1 in 9 people over the age of 65 diagnosed with AD (Rajan et al., 2021). *APOE*, the gene encoding apolipoprotein E, is the greatest genetic risk factor with *APOE4* increasing risk up to 15-fold compared to *APOE3* (Corder et al., 1993; Strittmatter et al., 1993), and *APOE4* is also associated with earlier age of AD onset (Corder et al., 1993; Locke et al., 1995; Bilgel et al., 2016; Polsinelli et al., 2023a). Females have increased AD risk compared to males (Brinton, 1999; Riedel et al., 2016). Importantly, there is evidence that the AD risk factors interact. For example, there is higher AD risk in female *APOE4* carriers, compared to male *APOE4* carriers (Farrer et al., 1997; Altmann et al., 2014), which may be pronounced at older ages (65–75 years of age) (Neu et al., 2017). Furthermore, in *APOE4* mice, behavioral impairments and AD pathology are worse in females compared to males (Raber et al., 1998, 2002; van Meer et al., 2007; Christensen et al., 2020). Therefore, it is important to understand how these risk factors interact to impact AD risk.

One way that AD risk factors may modulate cognition is by impacting amyloid beta (Aβ) accumulation as plaques, and/or neuroinflammation. In fact, *APOE4* is associated with greater levels of Aβ (Kok et al., 2009) and higher neuroinflammation (Reale et al., 2012; Ringman et al., 2012; Tzioras et al., 2019; Friedberg et al., 2020) compared to non-carriers. In addition, older women generally have higher levels of amyloid pathology compared to men (Oveisgharan et al., 2018) and dysregulated neuroinflammatory responses (Klein and Flanagan, 2016). These human data are recapitulated *in vivo* in young mice, as *APOE4* is associated with increased Aβ deposition and neuroinflammation, compared to *APOE3* mice (Youmans et al., 2012b; Rodriguez et al., 2014; Stephen et al., 2019, 2022). Therefore, evaluating the extent that *APOE* and sex impact pathology at older ages is important for advancing our understanding of how each risk factor contributes to AD pathogenesis across the lifespan.

The goal of our study was to understand the extent that *APOE* and sex interact with age to impact Aβ levels, neuroinflammation, neuronal density and behavior *in vivo*. To this end, we used EFAD mice as they express human *APOE3* (E3FAD) or *APOE4* (E4FAD) and overproduce Aβ42. Pathology and behavior were assessed in male and female EFAD mice at 6-, 10-, 14-, and 18-months-of-age using biochemistry, immunohistochemistry, and the Morris water maze test.

## 2. Materials and methods

### 2.1. Animals

All experiments follow the University of Illinois at Chicago Animal Care Committee protocols. EFAD mice express five familial AD (FAD) mutations and human *APOE*. Four groups of EFAD (5xFAD^+/−^/human *APOE*^+/+^) mice were used: male E3FAD, female E3FAD, male E4FAD, and female E4FAD mice as described previously (Youmans et al., 2012b). Mice from each of the four groups (male and female, E3FAD and E4FAD mice) were enrolled by age (i.e., 6, 10, 14, or 18 months euthanized together) as the breeding schedule permitted. Mice were ear-tagged during genotyping and investigators conducting experiments and data analysis were blinded for *APOE* genotype, sex, and age. Two cohorts of mice were used in this study (*n* = 13–25), one was used for biochemical and immunohistochemical analysis and the second for behavior analysis using Morris water maze.

### 2.2. Brain tissue harvest and processing

Mice were anesthetized via intraperitoneal injection with ketamine (100 mg/kg) and xylazine (5 mg/kg) and perfused transcardially with ~40 mL phosphate buffered saline with protease inhibitor cocktail. Then, the brains were removed and dissected at the midline to produce two hemi-brains, one each for immunohistochemical and biochemical analysis (Youmans et al., 2012b; Valencia-Olvera et al., 2023). The hemi for immunohistochemical analysis was drop-fixed in 4% paraformaldehyde for 24 h and then transferred to phosphate buffered saline containing 0.01% sodium azide until ready to section on a sliding microtome. Cortex was dissected from the hemi-brain for biochemical analysis, flash-frozen in liquid nitrogen and then stored at −80°C.

### 2.3. Immunohistochemical analysis

Serial sagittal brain sections (35 μm thick, 280 μm apart, ~0.24 mm – 3.44 mm lateral) from EFAD mice were immunostained for Aβ deposition, astrogliosis or microgliosis (Youmans et al., 2012b; Rodriguez et al., 2014; Valencia-Olvera et al., 2023) and stained for fibrillar amyloid deposition via Thio-S (see full list of antibodies and reagents used in this study in Supplementary Table S1). The stained sections were imaged at 10X magnification with a Zeiss Fluorescence microscope and analyzed for cortical area covered by MOAB-2, Thio-S, GFAP and Iba-1 in the cortex using ImageJ by investigators blinded to age, *APOE* genotype, and sex. Serial sagittal sections (three sections between ~0.72-and 1.80-mm lateral) from EFAD mice were stained for NeuN and neuron numbers quantification in layer 5 of the somatosensory cortex, was performed by a design-based stereology system (Stereo Investigator 9, MBF Bioscience, Williston, VT, USA) using an optical fractionator applying the N_v_ × V_ref_ method (West, 2002; Schmitz and Hof, 2007).

### 2.4. Biochemical analysis

Cortices were homogenized in 70% formic acid at 1 mL/150 mg brain tissue and mixed by end-over-end rotation at room temperature for 2 h with vortexing. Samples were then centrifuged (100,000 × *g*, 1 h at 4°C), and the formic acid-soluble fraction was neutralized (with 20 volumes of 1 M Tris base), aliquoted, and frozen at −80°C (Youmans et al., 2011). Total protein in the formic acid soluble extracts was quantified using the Bradford assay and formic acid-soluble Aβ42 was measured by ELISA following the manufacturer’s instructions (Youmans et al., 2011, 2012a,b). Cortical drebrin levels were measured using Western blotting as described previously (Valencia-Olvera et al., 2023).

### 2.5. Behavioral test: Morris water maze

All behavioral data were recorded and analyzed with ANY-maze software (Stoelting Co, Wood Dale, IL, USA). In the week prior to sacrifice, mouse behavior was tested using a modified Morris water maze protocol with acquisition trials consisting of 4 × 1 min trials/day for 5 consecutive days with latency to the platform recorded for each trial. A single probe trial was run on day 6 with the platform removed, and the readouts included latency to platform area, platform area crosses and latency to target quadrant (Liu et al., 2015; Thomas et al., 2017; Valencia-Olvera et al., 2023).

### 2.6. Data and statistical analysis

Supplementary File S1 is a word file containing Supplementary Table S1 and Supplementary Figures S1–S6. Supplementary File S2 is an excel file containing all raw data and statistical analysis tables including number of mice used for each experiment. Morris water maze acquisition phase data was analyzed by using repeated measure univariate general linear model for within subjects’ effects with the independent variables day, age, *APOE* and sex in SPSS (IBM SPSS Statistics for Macintosh, Version 29.0.1.1). All other statistical analyses were conducted using univariate general linear models for between subjects’ effects with the independent variables (age, *APOE* genotype, sex) and their interactions, followed by Bonferroni’s *post hoc* tests in SPSS. For all statistical tests, *p* < 0.05 was considered significant. All data are presented as scatter plots with the mean and standard error of mean (SEM). The *n* size range is indicated in Figure legends. Outliers were excluded using the ROUT method (*Q* = 5%). All data, detailed *n* sizes and statistical analysis are provided in Supplementary File S2.

## 3. Results

The goal of our study was to determine the extent to which age, *APOE* and sex modulates Aβ pathology, neuroinflammation and behavior *in vivo*. To this end, we used EFAD mice that overproduce Aβ42 (via 5xFAD mutations) and express human *APOE3* (E3FAD) or *APOE4* (E4FAD). EFAD mice were developed to identify how *APOE* modulates progression of AD-relevant pathology and behavior (Youmans et al., 2012b). Previous studies in EFAD mice have demonstrated that at younger ages (≤ 6 months), Aβ levels, reactive microglia and astrocytes are higher with *APOE4* compared to *APOE3* (Youmans et al., 2012b; Stephen et al., 2019, 2022), and that there are behavioral deficits in female *APOE4* mice (Liu et al., 2015). Therefore, we determined the extent that *APOE*, age and sex impact Aβ pathology, gliosis, and behavior in young (6 and 10 months), middle-aged (14 months) and old (18 months) EFAD mice.

### 3.1. *APOE* and sex impact Aβ pathology: E4FAD females > E4FAD males = E3FAD females > E3FAD

**Figure 1:**
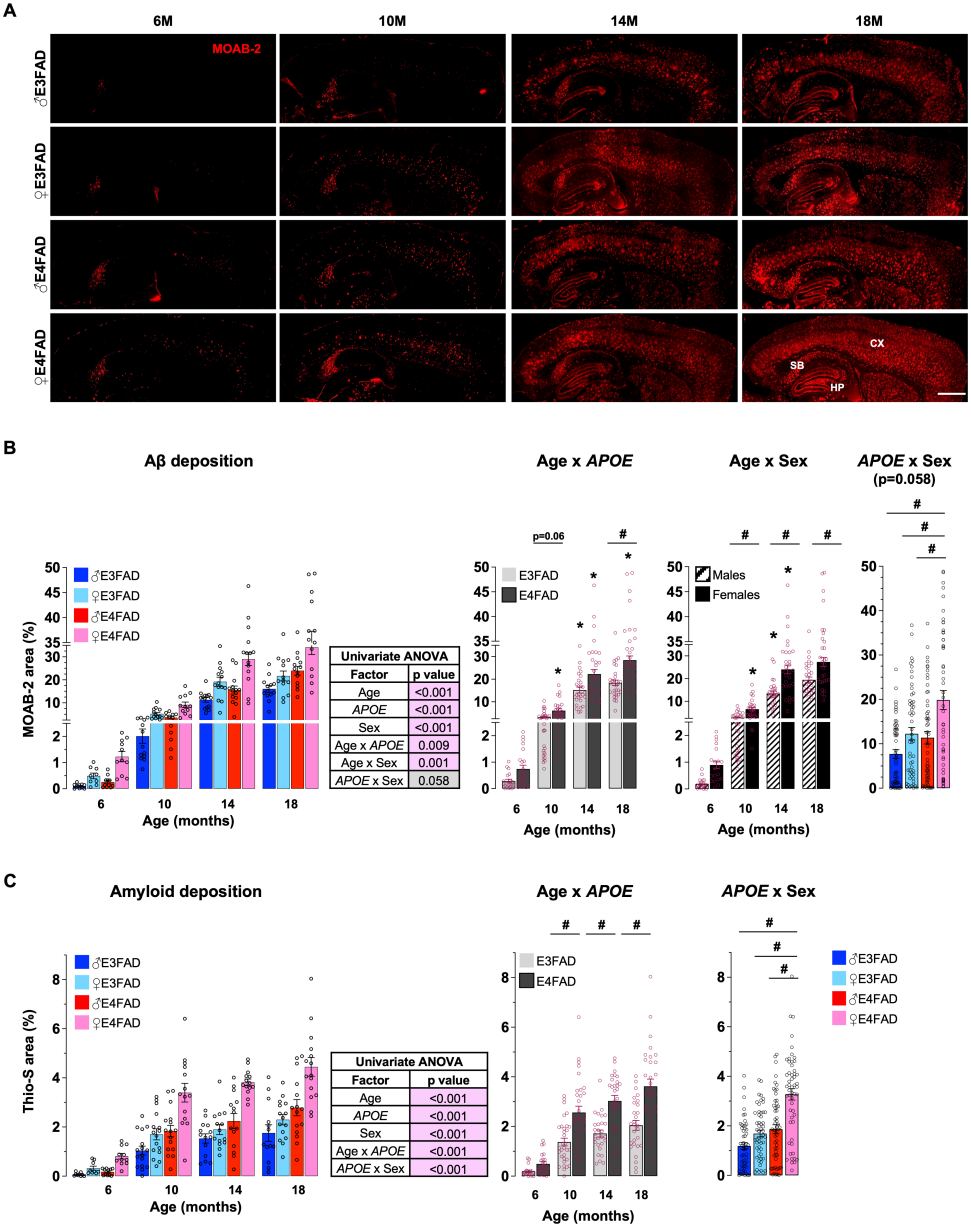
*APOE* and sex impact Aβ pathology: E4FAD females > E4FAD males = E3FAD females > E3FAD. Cortical extracellular Aβ deposits were assessed using histochemical analysis in male and female, E3FAD and E4FAD mice. **(A)** Brain sections were immunostained for Aβ using MOAB-2 (Red, scale bars: 1000 μm) and the percentage area quantified **(B)**. **(C)** Brain sections stained with Thio-S for fibrillar amyloid deposits (see Supplementary Figure S1 for representative images) were quantified for cortical amyloid burden. Data are expressed as mean ± S.E.M and analyzed by univariate general linear modeling followed by Bonferroni’s *post-hoc* analysis (*n* = 9–15, # *APOE*/sex difference within an age group, * vs. previous age within *APOE*/sex combination *p* < 0.05). See Supplementary File S2 for detailed *n* sizes and statistical analysis.

Extracellular Aβ in fibrillar amyloid deposits are used to diagnose AD and are a current therapeutic target (Zhang Y. et al., 2023). Although *APOE4* is associated with higher amyloid deposits in human and *in vivo*, the extent that *APOE* and sex modulate extracellular Aβ with age is unclear. Therefore, we initially evaluated cortical extracellular Aβ levels (MOAB-2 immunostaining for Aβ: Figure 1A) and fibrillar amyloid deposits (Thio-S staining: Supplementary Figure S1A) in male and female, E3FAD and E4FAD mice. We found that age interacts with *APOE* to modulate Aβ and fibrillar amyloid deposition (Figures 1B,C). This interaction was due to greater levels of Aβ at 10 and 18 months and amyloid deposits at 10, 14, and 18 months of age, but not at 6 months, in E4FAD mice compared to E3FAD mice. In general, amyloid deposition increased at every age (18 > 14 > 10 > 6 months) in E4FAD mice, whereas levels plateaued at ~10–14 months of age in E3FAD mice (Figures 1B,C). There was also an interaction between age and sex in modulating Aβ deposition, with greater levels in females compared to males at older ages (10, 14, and 18 months) but not at younger ages (6 months). For fibrillar amyloid deposition there was an *APOE* by sex interaction as levels were in the order E4FAD females > E4FAD males = E3FAD females > E3FAD males (Figure 1C), however there was no age x sex interaction (Supplementary Figure S1B). We also evaluated the levels of formic acid soluble Aβ42 in the cortex (Supplementary Figure S2). In general, the pattern of insoluble Aβ42 levels appears like extracellular Aβ deposition, however the levels were more varied. Thus, at the statistical level formic acid Aβ42 levels were higher with *APOE4* compared to *APOE3* and in females vs. males at older ages (10–18 months).

Collectively these data support that female sex and *APOE* have a strong effect on extracellular Aβ deposition. That interaction results in highest fibrillar amyloid deposits in female *APOE4* carriers, equivalent levels in female *APOE3* and male *APOE4* carriers, and lowest levels in male *APOE3* carriers at older ages.

### 3.2. *APOE* and sex affect neuroinflammation: E4FAD females > E3FAD females ≥ E4FAD males > E3FAD males

**Figure 2:**
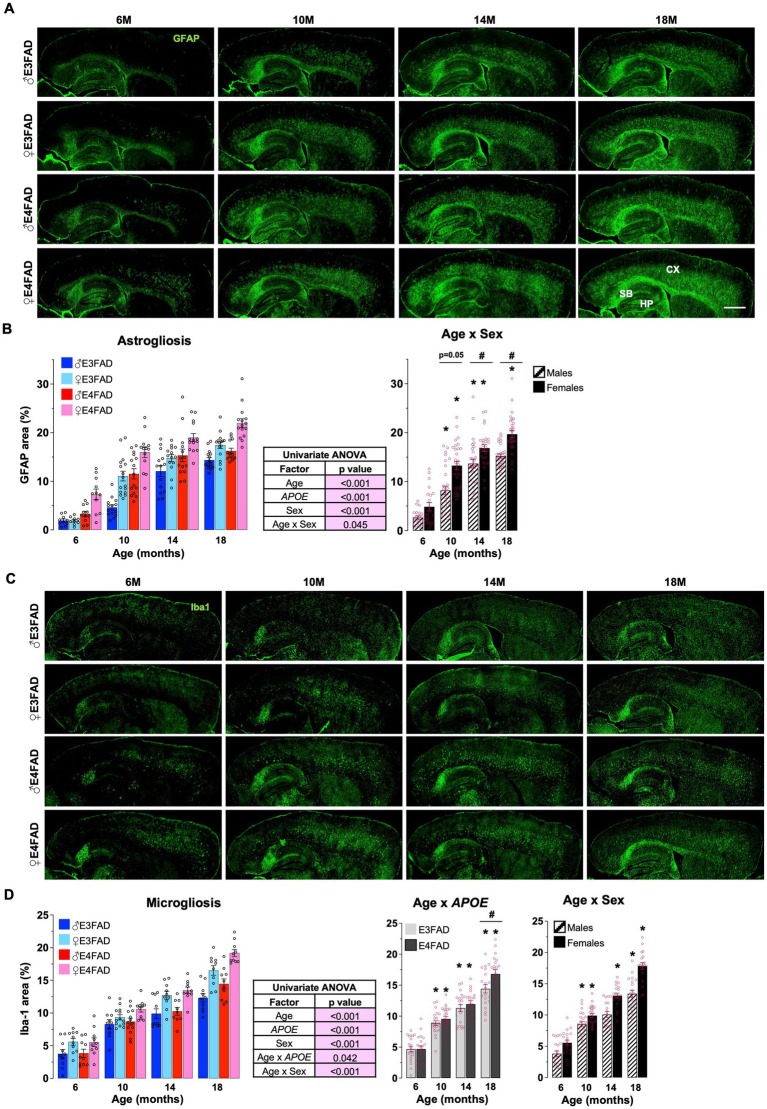
Sex impact neuroinflammation: E4FAD females > E3FAD females > E4FAD males > E3FAD males. Reactive astrocytes and microglia were assessed using immunohistochemical analysis in male and female, E3FAD and E4FAD mice. **(A)** Brain sections were immunostained for astrogliosis using GFAP (green, scale bars: 1000 μm) and the percentage area of cortex covered was quantified **(B)**. **(C)** Reactive microglia in brain sections were immunostained for using Iba-1 (green, scale bars: 1000 μm) and the percentage area of cortex was quantified **(D)**. Data are expressed as mean ± S.E.M and analyzed by univariate general linear modeling followed by Bonferroni’s *post-hoc* analysis (*n* = 9–15, # *APOE*/sex difference within an age group, * vs. previous age within *APOE*/sex combination *p* < 0.05). See Supplementary File S2 for detailed *n* sizes and statistical analysis.

*APOE4* is associated with higher inflammatory responses in younger EFAD mice (Vitek et al., 2012; Rodriguez et al., 2014; Stephen et al., 2022) and in humans (Reale et al., 2012; Ringman et al., 2012; Tzioras et al., 2019; Friedberg et al., 2020). Therefore, we next evaluated the extent that *APOE*, age and sex impacted levels of reactive astrocytes (GFAP: Figure 2A) and microglia (Iba-1: Figure 2C) via immunostaining, in male and female, E3FAD and E4FAD mice. We found that GFAP coverage was higher with *APOE4* compared to *APOE3*, and for Iba-1 there was interaction between *APOE* and age with higher coverage in E4FAD mice compared to E3FAD mice at 18 months of age with no effect at other ages (there were also no interactions between age and *APOE* or *APOE* and sex, Supplementary Figure S3A). As for extracellular Aβ, we found that age interacts with sex to modulate both astrogliosis and microgliosis. This interaction was due to significantly greater GFAP and Iba-1 coverage in the cortex of female mice between 10 and 18 months (Figures 2B,D). Furthermore, both percentage area covered by GFAP and Iba-1 increased at every age (18 > 14 > 10 > 6 months) in female mice, whereas levels plateaued at ~10–14 months of age in male mice (Figures 2B,D). Overall, these results support that neuroinflammation measured as gliosis was significantly impacted by female sex and *APOE*.

### 3.3. Female E4FAD mice have early behavioral deficits in acquisition phase of Morris water maze

**Figure 3:**
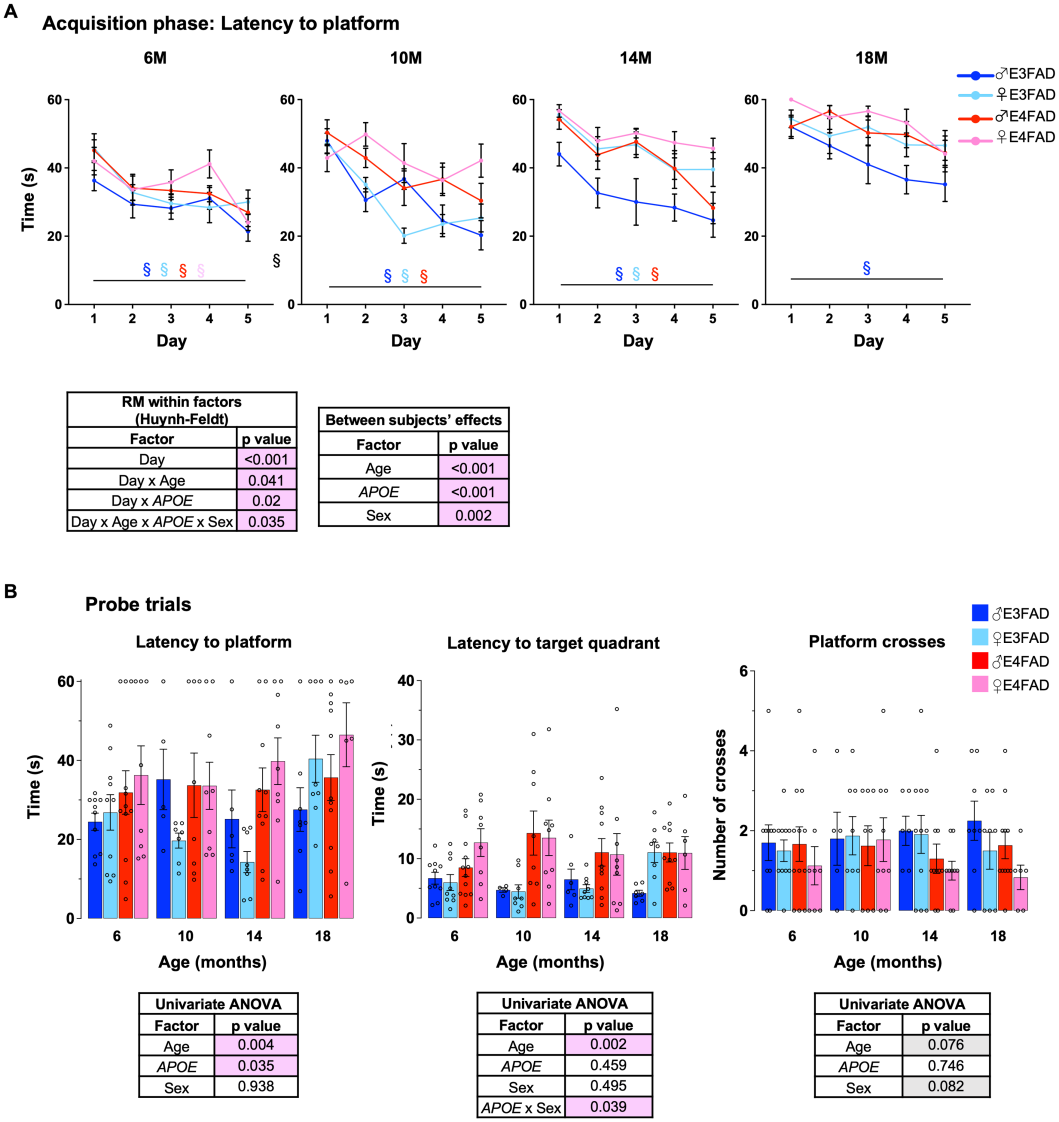
Female E4FAD mice have early behavioral deficits. Learning and memory were assessed via Morris water maze. **(A)** EFAD mice were trained to locate the location of a platform over 5 days (acquisition phase) and **(B)** the ability to remember the location of the platform 24 h after the last training day (probe trial). Data are expressed as mean ± S.E.M. Latency to platform during acquisition phase was analyzed by repeated measure general linear modeling followed by Bonferroni’s *post-hoc* analysis (*n* = 9–15, § day 1 vs. day 5 matched by mouse group color, *p* < 0.05). Probe trial measures were analyzed by univariate general linear modeling followed by Bonferroni’s *post-hoc* analysis (*n* = 9–15, # *APOE*/sex difference within an age group, * vs. previous age within *APOE*/sex combination *p* < 0.05). See Supplementary File S2 for detailed *n* sizes and statistical analysis.

AD is associated with changes in cognitive trajectory in humans (Jack et al., 2015; Rasmussen et al., 2018) and behavioral deficits in FAD mice (Webster et al., 2014; Jankowsky and Zheng, 2017) including EFAD mice (Liu et al., 2015; Marottoli et al., 2017; Thomas et al., 2017). Therefore, we evaluated the effect of age, *APOE* and sex in EFAD mice using a modified Morris water maze protocol. It is important to note that our protocol did not include a visible platform. Therefore, we cannot discern whether the swim speeds differ among the four cohorts of mice with age, which in turn may affect their performance in the water maze test. In the acquisition phase, we found that there was an interaction among age, *APOE*, sex and training day. This interaction was due to a few factors, one of which was differences among groups at each age. When comparing training day 1 to 5, all the four groups of mice learned the location of the platform 6 months of age (Figure 3A: 6 M) (i.e., no effect of *APOE* or sex). However, at 10-and 14-months of age, female E4FAD mice did not improve in performance from day 1 to day 5 (Figure 3A: 10 M and 14 M). Furthermore, at 18 months of age, only male E3FAD mice showed a significant latency reduction from day 1 to day 5 (Figure 3A: 18 M). These data supported that female E4FAD mice had impairments in acquisition at an earlier age than other groups. Infact, when the day 5 latencies at 6 months of age were compared to other ages, we found deficits at 10 months in E4FAD female mice, at 18 months in E3FAD females and E4FAD males, with no changes in E3FAD males (Supplementary Figure S4A). We also evaluated memory 24 h after the last training day using a single probe trial. We found that most probe measures (latency to platform, latency to target quadrant and platform crosses) were impacted by *APOE* (Figure 3B). However, age, *APOE* and sex did not affect time spent in target quadrant during the probe trials (Supplementary Figure S4B). Also, there were no interactions among age, *APOE* and sex for the probe trial measures (Supplementary Figures S4C–F). Importantly, the latency to platform was independently modulated by *APOE* due to a greater latency to find the platform area and target quadrant in E4FAD compared to E3FAD mice.

We next assessed neuronal density (NeuN) in layer 5 of somatosensory cortex in male and female, E3FAD and E4FAD mice (Supplementary Figure S5A). Similar to the probe trial measures for memory, we found that neuronal density (Supplementary Figure S5B) and Drebrin levels (Supplementary Figure S5C) were impacted by age (lower with age) and *APOE* (*APOE4* > *APOE3*). Collectively, our data support that E4FAD females demonstrated earlier behavioral deficits and loss of neurons at 6–10 months of age, compared to the other groups.

## 4. Discussion

### 4.1. Aβ deposition

Aβ deposition as plaques is one of the main AD pathologies (Rodrigue et al., 2009; Ruan and Sun, 2023; Suemoto and Leite, 2023). As a well-established AD risk (Kawas et al., 2000; Farfel et al., 2019), age has been proposed to modulate Aβ deposition. However, amyloid deposits occur in about 20% of cognitively normal elderly subjects and therefore may also be modulated by other AD risk factors. *APOE* has a pronounced impact on Aβ deposition. Aβ levels are higher with *APOE4* in human (Villemagne et al., 2011; Chiotis et al., 2015; Jack et al., 2015; Resnick et al., 2015; Mishra et al., 2018; Ba et al., 2019; Baek et al., 2020; Therriault et al., 2020, 2021; Cacciaglia et al., 2022) and mouse studies (Youmans et al., 2012b; Moser and Pike, 2017; Montagne et al., 2021). Sex is also thought to impact Aβ deposition. The independent effect of sex in modulating Aβ deposition is conflicting in humans (Jack et al., 2015; Ba et al., 2019; Therriault et al., 2020, 2021; Cacciaglia et al., 2022), potentially due to variable hormone levels, disease stage, co-morbidities and other lifestyle factors. However, in FAD mice, females have greater Aβ pathology compared to males (Wang et al., 2003; Li et al., 2016; Dennison et al., 2021; Forner et al., 2021; Marazuela et al., 2022; Sil et al., 2022). Our data agree with *in vivo* and human studies that the combination of *APOE4* and female sex results in highest levels of Aβ deposition compared to other groups (Figure 1; Supplementary Figure S2). Interestingly, our data also demonstrate that Aβ pathology is equivalent in female E3FAD mice and male E4FAD mice. These novel findings raise the possibility that female sex and *APOE4* induce a similar impact on Aβ pathology. These findings support that female sex may be an important factor for enrolling in preclinical/clinical trials targeting Aβ (Shi et al., 2022).

### 4.2. Neuroinflammation

Neuroinflammation is also an important AD pathology, often defined as levels of reactive astrocytes and microglia (glia) in the brain (Streit et al., 2004). In general, in AD patients higher levels of reactive glia are thought to occur early in disease progression (Hoozemans et al., 2011), in females compared to males (Duarte-Guterman et al., 2020; Lynch, 2022) and with *APOE4* (Reale et al., 2012; Ringman et al., 2012; Tzioras et al., 2019; Friedberg et al., 2020). However, the interaction among age, *APOE* and sex on reactive glia has yet to be characterized in AD patients, and therefore FAD mouse models are currently being used to address this issue. *In vivo* studies demonstrate that there is greater neuroinflammation in females compared to males in FAD mice (Hou et al., 2015; Schmid et al., 2019; Guillot-Sestier et al., 2021; Mifflin et al., 2021; Sil et al., 2022). This sex effect is pronounced with *APOE4* in young mice (Stephen et al., 2019, 2022). We have extended these findings to older mice and demonstrate that the combination of *APOE4* and female sex results in the highest levels of reactive glia with age. In addition, our data revealed that in general, female E3FAD mice have greater neuroinflammation compared to male E4FAD mice. Collectively, our data demonstrates that targeting neuroinflammation may be an attractive therapeutic approach for females AD patients.

### 4.3. Behavior

AD is typically characterized by memory loss, and a decline in overall cognitive function (Rao et al., 2023). Several studies demonstrate that with age, cognitive decline is accelerated in AD patients (Rosselli et al., 2000; Conde-Sala et al., 2013; Zhao et al., 2014; Pan et al., 2021) and FAD mice (Middei et al., 2006; Ferguson et al., 2013; Webster et al., 2013; Olesen et al., 2016; Forner et al., 2021; Locci et al., 2021). Among AD patients, cognitive decline is pronounced in *APOE4*, compared to *APOE3* carriers (Emrani et al., 2020; Gharbi-Meliani et al., 2021; Qian et al., 2021, 2023; Polsinelli et al., 2023b). This effect is mirrored in FAD mice in the presence of *APOE4*, compared to *APOE3* in young (Thomas et al., 2017) and old (Montagne et al., 2021) mice. In addition, sex impacts cognitive decline in AD patients and memory deficits in mice. In general, women display poorer cognitive profiles compared to men at the same stage of AD (Subramaniapillai et al., 2021) and is mirrored in female mice compared to male FAD mice (Poon et al., 2023), also reviewed in Li and Singh (2014) and Laws et al. (2016). In this study, we evaluated the combined effect of age, *APOE* and sex in Morris water maze performance and found that female E4FAD mice had earlier behavioral deficits during acquisition phase of Morris water maze compared to other groups (Figure 3A). Consistent with other readouts, *APOE3* female and *APOE4* male had similar behavioral deficits with age.

### 4.4. Limitations

There are some limitations due to the nature of our design. One issue is that we are limited in the extent that we can conclude *APOE*, sex and age impacted all aspects of AD pathology and behavior. Future studies could incorporate more detailed evaluation of Aβ production, clearance, and degradation pathways, the full neuroinflammatory phenotype, other AD-prevalent pathologies such as vascular function and metabolism, neuronal function, and a full battery of behavioral tests in the cognitive domain. Furthermore, it is important to validate our findings in other models that incorporate human *APOE* and human *APP*. For example, human APP knock in mice have been crossed with *APOE* targeted replacement mice, and data supports greater behavioral deficits and Aβ pathology with *APOE4* (Holden et al., 2022). In addition, understanding the effect *APOE2* genotype which is protective for AD risk is important, with the caveat that these mice have Type III hyperlipoproteinemia (Sullivan et al., 1998). Our study raises an important discussion of the possible underlying mechanisms of how *APOE4* and female sex impact AD pathology and cognition. For example, structural differences between apoE isoforms may modulate Aβ levels, glial reactivity, and neuron function [reviewed in Hunsberger et al. (2019), Martinez-Martinez et al. (2020), Raulin et al. (2022), Yang et al. (2023), and Zhang L. et al. (2023)]. As for female sex, the increase in AD risk/pathology has been attributed to loss of sex hormones with age particularly at menopause in humans (Rocca et al., 2011; Levine et al., 2016; Jett et al., 2022; Mishra et al., 2022), which is recapitulated in ovariectomized mice (Ge et al., 2020; Sanchez et al., 2023). Although mice do not have the typical menopausal symptoms as humans, there are age-dependent changes in hormonal levels, which could impact AD-pathology and cognition in mice. Indeed, we observed that the number of mice in proestrus/estrus decrease with age in female EFAD mice (Supplementary Figure S6). In addition, there may be X chromosome-mediated modulation of AD risk (Guo et al., 2022) may partly explain the sex-biased differences in AD. Furthermore, our study has also raised questions for future research including: What aspect of AD pathology is proximal to behavior changes in EFAD mice? What is the effect of *APOE3/4* genotype on AD pathology and the interaction with sex? Does the impact of sex and *APOE* on pathology modify responses to therapeutic treatments?

### 4.5. Conclusion

Our data support that the combination of female sex and *APOE4* result in the greatest levels of Aβ pathology, neuroinflammation and behavioral impairments. In addition, we propose that the effect of female sex is analogous to the presence of *APOE4* for AD pathology. Through reviewing previous published *in vivo* and human data, we believe that this important concept may have been overlooked (Holland et al., 2013; Huynh et al., 2019; Stephen et al., 2019, 2022; Cho et al., 2021; Montagne et al., 2021; Palmer et al., 2022). The female sex and *APOE4* equivalency could therefore impact the optimal treatment window for therapeutics aimed at preventing AD through targeting Aβ and/or neuroinflammation.

## Data availability statement

The original contributions presented in the study are included in the article/Supplementary material, further inquiries can be directed to the corresponding author.

## Ethics statement

The animal study was approved by University of Illinois Animal Care Committee. The study was conducted in accordance with the local legislation and institutional requirements.

## Author contributions

DB: Writing – original draft, Writing – review & editing, Data curation, Formal analysis, Investigation, Project administration, Supervision. AV-O: Data curation, Investigation, Supervision, Writing – review & editing. ZI: Writing – review & editing, Methodology. CM: Writing – review & editing, Methodology. AH: Writing – review & editing, Methodology. TP: Writing – review & editing, Methodology. JY: Methodology, Writing – review & editing. ML: Conceptualization, Funding acquisition, Project administration, Supervision. LT: Writing – review & editing, Writing – original draft.

## In memoriam

This manuscript is in memory of Dr. Mary Jo LaDu, who recently tragically passed away. She will be missed by all.
