# Supplementary material for: APOE genotype and sex modulate Alzheimer’s disease pathology in aged EFAD transgenic mice
Source: Front Aging Neurosci. 2023 Oct 31;15:1279343. doi: 10.3389/fnagi.2023.1279343 (PMC10644540; doi:10.3389/fnagi.2023.1279343)
Supplement: Supplementary file 1 [file Data_Sheet_1.docx]

Supplementary Material

*APOE* genotype and sex differentially influence the extent of Alzheimer’s disease pathology in aged EFAD transgenic mice

Deebika Balu^1^, Ana C. Valencia-Olvera^1^, Zarak Islam^2^, Clare Mielczarek^1^, Allison Hansen^3^, Tamara M Perez Ramos^4^, Jason York^1^, Mary Jo LaDu^1^, Leon Tai^1, *^

^1^Department of Anatomy and Cell Biology, University of Illinois at Chicago, Chicago, Illinois, USA

^2^University of Illinois College of Medicine, Chicago, Illinois, USA

^3^University of Illinois College of Medicine, Peoria, Illinois, USA

^4^School of Medicine, St. George’s University, Grenada, West Indies

*** Correspondence:** Leon Tai: leontai@uic.edu

## Supplementary Table 1

## Supplementary Figure 1

## Supplementary Figure 1. Representative images of cortical fibrillar amyloid deposits were assessed using histochemical analysis in male and female, E3FAD and E4FAD mice (Green, scale bars: 1000µm) (A). Age does not interact with sex to impact amyloid pathology (B). Data are expressed as mean +/- S.E.M and analyzed by univariate general linear modelling followed by Bonferroni’s *post-hoc* analysis (n=9-15). See Supplementary file 2 for detailed *n* sizes and statistical analysis.

## Supplementary Figure 2

## Supplementary Figure 2. Insoluble Aβ42 levels are higher in E4FAD mice compared to E3FAD, and in females vs. males at older ages (10-18 months). Aβ42 was measured in formic acid-soluble fraction of the cortex in male and female, E3FAD and E4FAD mice. Data are expressed as mean +/- S.E.M. All data analyzed by univariate general linear modelling followed by Bonferroni’s *post-hoc* analysis (n=9-15, # *APOE*/sex difference within an age group, * vs. previous age within *APOE*/sex combination *p*<0.05). See Supplementary file 2 for detailed *n* sizes and statistical analysis.

## Supplementary Figure 3

## Supplementary Figure 3. Age does not interact with *APOE* to impact astrogliosis (A). APOE and sex does not interact to modulate astrogliosis (A) or microgliosis (B). Data are expressed as mean +/- S.E.M and analyzed by univariate general linear modelling followed by Bonferroni’s *post-hoc* analysis (n=9-15). See Supplementary file 2 for detailed *n* sizes and statistical analysis.

## Supplementary Figure 4

## Supplementary Figure 4. E4FAD females demonstrated behavioral deficits at 10 months compared to other groups (A). Latency to platform on day 5 was analyzed in male and female, E3FAD and E4FAD mice. Time spent in target quadrant during the probe trial did not differ among the mice across ages (B). Time spent in target quadrant (quadrant that had the platform) during probe trial was analyzed in male and female, E3FAD and E4FAD mice. There were no interactions between age and *APOE*/ sex or between *APOE* and sex for probe trial measures including latency to platform (C), latency to target quadrant (D), platform crosses (E) and time spent in target quadrant (F). Data are expressed as mean +/- S.E.M. All data analyzed by univariate general linear modelling followed by Bonferroni’s *post-hoc* analysis at each age (n=9-15, # *APOE*/sex difference within an age group, * vs. previous age within *APOE*/sex combination *p*<0.05). See Supplementary file 2 for detailed *n* sizes and statistical analysis.

## Supplementary Figure 5

**Supplementary Figure 5**. **Neuronal cell density and neuron-specific proteins decreased with age**. Brain sections were immunostained with NeuN to label neurons (Green, scale bars: 1000µm) (**A**) and their numbers were assessed by counting NeuN+ cells in the layer 5 of somatosensory cortex of male and female, E3FAD and E4FAD mice (**B**) as layer 5 of the somatosensory cortex, compared to other superficial layers show significant Aβ deposition in EFAD mice. For the analysis of NeuN+ cells three serial sections between ~ 0.72- and 1.80-mm lateral were taken for stereological analysis. The layer 5 of somatosensory cortex was traced at low magnification (5x), and all cell counts were performed at high magnification (63x; Zeiss AX10 microscope; Carl Zeiss Ltd., Hertfordshire, England). The sampling parameters were as follows: counting frame width X = 75 µm, counting frame height Y = 75 µm, sampling grid X = 200 µm, Y = 200 µm, dissector height Z = 26 µm, and guard zone distance = 1-2 µm. Drebrin levels were measured in male and female, E3FAD and E4FAD mice by Western blot and normalized to β-actin (Top: representative Western blot image) (**C**). Data are expressed as mean +/- S.E.M and analyzed by univariate general linear modelling. See supplementary file 2 for detailed *n* sizes and statistical analysis.

## Supplementary Figure 6

## Supplementary Figure 6. Age affects estrous stage distribution in female EFAD mice. Before euthanasia, the estrous stage of every female mouse at 6, 10, 14 and 18 months of age, was assessed by vaginal cytology. Estrous stage distribution data is expressed as percentage of mice in either proestrus/estrus or metestrus/diestrus for female E3FAD (A) and E4FAD (B) mice across ages.
